# Power-assisted pedicle screws placement: Is it as safe and as effective as manual technique? Narrative review of the literature and our technique

**DOI:** 10.1007/s12306-021-00714-x

**Published:** 2021-05-29

**Authors:** C. Faldini, G. Viroli, M. Fiore, F. Barile, M. Manzetti, A. Di Martino, A. Ruffilli

**Affiliations:** Department of Biomedical and Neuromotor Science—DIBINEM, 1St Orthopaedic and Traumatologic Clinic, IRCCS Istituto Ortopedico Rizzoli, University of Bologna, Bologna, Italy

**Keywords:** Spine surgery, Scoliosis, Pedicle screws, Power tools, Posterior spinal fusion

## Abstract

Pedicle screws are the gold standard in spine surgery, allowing a solid tricolumnar fixation which is unmatched by hooks and wires. The freehand technique is the most widely adopted for pedicle screws placing. While freehand technique has been classically performed with manual tools, there has been a recent trend toward the use of power tools. The aim of this review is to summarize and expose potential risks and advantages of power pedicle screws placing. The literature showed that the use of power tools offers an acceptable safety profile, comparable to manual technique. With an adequate training, the power technique may speed up the screw placing, reduce the fluoroscopy time and the physical stress to the spine surgeon. Regarding differences in pull-out strength between power and manual techniques, the literature is still uncertain and inconsistent, both in clinical and preclinical studies. The choice between the use of power and manual freehand pedicle screws placing is still based on the surgeon’s own preference.

## Introduction

Pedicle screws were first introduced by Boucher [1] in the 1950s and later popularized by Roy Camille [2, 3] in the 1960s as a mean for achieving an optimal vertebral fixation. Compared to hooks and wires, they were biomechanically superior [4], allowed sacral fixation, provided fixation even when a laminectomy was performed, and did not require the violation of the canal. Initially, they were mostly adopted in lumbar spine and only later they were introduced in the thoracic spine, mainly due to a safety concern: in fact, thoracic pedicles are thinner in diameter compared to lumbar ones and they also lie just adjacent to vital structures whose injury may lead to irreversible consequences. Moreover, pediatric patients, with smaller pedicles, and deformity cases, with dysplastic pedicles, pushed the challenges of pedicle screws insertion even further. Despite that, pedicle screws have now become the gold standard both in thoracic and lumbar spine because of the solid tricolumnar fixation they provide: they allow to exert powerful correction forces and to achieve strong stability, yet still the margin of error in their placement remains small. For this reason, there is continuous interest in refining their placement technique, to increase accuracy and reproducibility of vertebral instrumentation, providing a safer and stronger fixation. Recent literature has been focusing on freehand fluoroscopy-assisted [5], computed tomography navigation-guided [6, 7] and robot-assisted techniques [8, 9]. While the above-mentioned alternatives remain attractive, conventional freehand technique remains the most popular choice.

Pedicle screws placing, regardless of the technique adopted, follows these fundamental steps: hole preparation, palpation, hole tapping, re-palpation, screw insertion. Classically, the traditional freehand technique has been performed with manual tools: a Gearshift for hole preparation, a manual tap for tapping and a manual screwdriver for insertion. While the principles of freehand pedicle screws placing have remained the same (perforation, tapping, insertion), there has been a recent trend toward the use of power tools, considering some critical issues. Firstly, it has been demonstrated that spine surgeons are at greater risk of musculoskeletal overuse injuries [10]. Secondly, it has been reported that orthopedic surgeons suffer excessive exposure to radiation due to the use of fluoroscopy [11–14].

The aim of this study is to review the literature about freehand power pedicle screws placing technique, assessing associated risks and benefits compared to the manual technique.

## Pull-out strength

The aspect that could really make a difference in clinical outcomes is the pull-out strength and the consequent risk of screw failure. In this regard, there are uncertain and inconsistent information regarding any difference between power and manual technique.

George et al. [15] performed a biomechanical comparison between transpedicular screws implanted into drilled and probed holes. In each of the eight thoracolumbar human cadaveric vertebrae they used, a preparation hole was made through one pedicle with a 4 mm drill bit and through the contralateral pedicle with a probe. They did not report any statistically significant difference in the pull-out strength between the two techniques, with a slightly superior (1.4%) strength for the probed screws. However, it must be noted that the 4 mm drill bit appears far bigger than the drill bit sizes that are commonly used in surgical practice, and this, by reducing the difference between screw and tract diameter, may reduce the pull-out strength.

Abraho et al. [16] registered similar results in terms of insertion torque and pull-out strength between drill, sharp probe and pointed probe screws insertion in a swine vertebral model. The only situations where a statistically significant difference was noted in the insertion of 4.2 mm screws were: a greater torque when making a 3.4 mm pilot hole with a drill compared to the sharp probe; a greater pull-out strength when making a 1.6 mm pilot hole with a drill compared to the pointed probe; a smaller pull-out strength when making a 2.8 mm hole with a drill compared to a sharp probe.

Oikonomidis et al. [17] carried a biomechanical study to compare the anchoring strength of pedicle screws in osteoporotic vertebrae using a 3.2 mm drill bit versus a curved thoracic probe. They subjected their specimens to cranio-caudal cyclic loading, simulating a more physiological condition such as axial forces transmitted to the pedicle screws compared to the study conducted by George et al. [15], in which a simple pull-out strength test was performed. The probe showed higher tear-out force and higher number of load cycles until loosening than the 3.2 mm drill bit, but without statistical significance.

Considering the wide spread of pedicle screws and of the use of power tools in other districts, it is surprising that the first retrospective study about this topic has been written by Seehausen et al. [18] only in 2015. They used a 2 mm drill bit to create the tract, and then they expanded it with a 3.22 mm bit; they then inserted the screw without any prior tap. They included 442 cases and found that, after 2 years of follow-up, the screws placed using the manual method failed and required revision or removal 5.9 times as frequently as screws placed with the power method (0.8 vs. 0.1%; *p* = 0.024). When including all patients, regardless of the follow-up length, the difference in failure rate increased and the revision rate owing to screw failure was also statistically higher in the manual group (*p* = 0.022). However, it needs to be underlined that constructs in the power group got a higher screw density, which could itself lead to a reduction in the mechanical complications rate and represent a possible bias of the study.

Theoretically, the probe insertion leads to a compaction of the cancellous bone around the probe [17, 19], creating a more solid anchorage for the screw compared to the drill bit, which conversely may lead to bone removal. Despite that, preclinical studies showed comparable [15–17] fixation strength between the two. Conversely, a clinical study by Seehausen et al. [18] showed a significantly lower failure rate for power-assisted placed screws. One possible explanation for that is the reduction in the wobble phenomenon, which may improve the bone-screw interface, but this point requires further investigation, hopefully from both clinical and preclinical studies. Moreover, given the variability that currently exists in the technique (drill bit diameter, choice of tract tapping or not, method of tapping) further research is needed in order to create more homogeneity and obtain more comparable results.

## Safety

When talking about pedicle screws placing, safety and reliability are a major concern, considering the possible catastrophic consequences that a misplaced screw may have.

Allen et al. [20] carried a cadaveric study in order to compare the safety of pedicle screw placement in the thoracolumbar spine by resident surgeons with gearshift versus drill technique. There was no overall difference in violations comparing the gearshift technique (49.5%, 51 total, 37 critical, 14 noncritical) with drill technique (50.5%, 52 total, 33 critical, 19 noncritical). This may suggest that residents could be trained to use either one of the proposed techniques and ultimately learn how to instrument safely and accurately [20].

In terms of accuracy, a reduced screw wobble (where wobble is defined as the deviation of the screw from its intended trajectory) has been recorded with power screw insertion by Skaggs et al. [21] and Mahajan et al. [22]. In particular, Skaggs et al. in a cadaveric study found that power technique reduces wobble length by 95% and wobble area by 59%, 84%, and 59% for matched 35 mm, 40 mm, and 45 mm screws (*p* < 0.05) [21]. Looking at these findings, it seems inevitable that a certain amount of wobble would occur during the change of grip when using manual instruments; on the contrary, when using a power tool, it is easier to maintain a consistent and accurate trajectory. This may have important implications since the wobble phenomenon may lead to erosion of the bone surrounding the defined pedicle trajectory, leading also to a lower screw purchase and reduced screw pull-out strength.

In their study comparing manual versus power-assisted screw placement on 442 cases, Seehausen et al. [18] found only one direct injury occurred, in the power-assisted group, a minor hemothorax. However, statistical analysis revealed a comparable risk of injury per screw between the two groups, confirming the safety profile of the technique. However, the study got some possible important biases: firstly, the power-assisted cohort got a shorter mean follow-up, which could lead to complications miss; secondly, screws in the manual group were placed before screws in the power group, therefore any difference may have been determined by the increasing experience of the surgeon.

Yan et al. [23] designed a prospective randomized controlled trial to compare full power-assisted (FPA) and manual technique in 105 AIS cases. In their FPA technique, they adopted a 2.0 mm drill bit, with a slow rotation (2–3 rotations/s) to create the tract, followed by the synchronous expanding and tapping with a 3.2 mm drill bit; finally, the screw was inserted using the same power drill. A significantly shorter screw insertion phase and screw placement time were noted in the FPA group (*p* < 0.0001). They recorded a non-significative trend toward decreased operative times and blood loss in the FPA cohort. Most importantly, no safety differences have been recorded, with comparable misplacement rates and patterns between the two techniques. This is the most crucial aspect, especially in AIS, since pedicle screw placing may be particularly challenging due to rotation and dysplasia of the pedicles. Interestingly, the rate of anterior cortex perforation was unexpectedly lower in the FPA group (10.6 vs. 15.3%), yet without statistical significance. The strengths of this study are its prospective RCT design and the homogeneity of patients (only AIS) and of constructs (instrumented levels, screw density). One possible confounding factor is the fact that surgeries were performed by three different surgeons.

Kojima et al. [24] performed a comparative study between power and manual technique in percutaneous pedicle screw placement. They used a manual cannulated probe for guidewire insertion, after which the manual method was used to insert the pedicle on one side and the power tool on the other side. The power method proved to be 2.5 × faster than the manual method, without any difference in placement accuracy at the postoperative CT assessment. Kojima et al. also suggested a possible advantage of power tools in percutaneous screws placing: during the procedure, rarely, the guidewire can bend when the screw is manually insert, deforming the screw tip. This may be due to a slight slip of the wrist or forearm during manual placement, so in this regard power tools may be advantageous.

## Fluoroscopy time

The use of fluoroscopy, although it improves the confidence in pedicle screws placing, poses the spine surgeon, and only marginally the patient, to radiation-related risks [11–14].

Seehausen et al. [18] found that power method was associated with shorter fluoroscopy time (37.44 ± 15.70 s vs. 55.40 ± 32.53 s; *p* < 0.001), resulting in a reduced radiological risk both for the surgeon and patients.

## Surgeon musculoskeletal overuse injuries

As already stated, a recent survey [10] of the SRS showed that spine surgeons are at greater risk of overuse injuries, in particular of the hand, wrist, shoulder and cervical spine.

In this regard, the only study focusing on the possible role of power tools in screw placement was carried out by Skaggs et al. [25]. They showed that manual pedicle preparation results in significantly increased muscle exertion of the biceps compared to power pedicle preparation. While this may seem a quite predictable fact, it may have unpredictable implications. On the one hand, it may reduce the risk of musculoskeletal injuries for spine surgeons. On the other hand, working with a more relaxed musculature may allow a better control of the trajectory and a more refined feel. In other words, all the surgeon has to do is to keep a well relaxed arm and focus only on the direction of the tract and on the tactile feedback of the drill, rather than care about exert enough force to create a tract.

## Our experience: technical tips and tricks, critical aspects

At our division, pedicle screws are routinely placed using power-assisted technique since more than 10 years [26–28]. Surprisingly, there is a paucity of studies in the literature about this technique, especially if one thinks that almost every screw insertion in orthopedic surgery is nowadays performed with the aid of power tools. Our freehand technique provides the use of power tools for each step of the pedicle screw placing. Antero-posterior and lateral fluoroscopic images are only obtained after all screws are placed, to verify their correct position.

Firstly, a careful identification of correct entry points should be carried on, using anatomical landmarks for each section of the spine. In the lumbar spine, the entry point is located at the junction between the pars interarticularis and the transverse process immediately lateral to the mammillary process or at the bisection of a vertical line through the facet joints and a horizontal line through the transverse process [29]. In the thoracic spine, it is identified in the middle of the triangle formed by the pars interarticularis, the lower border of the superior articular facet and the medial border of the transverse process [29]. Passing from T12 to T7, the entry points are more medial and cephalad, while above T7 are more lateral and caudal [30].

**Fig. 1:**
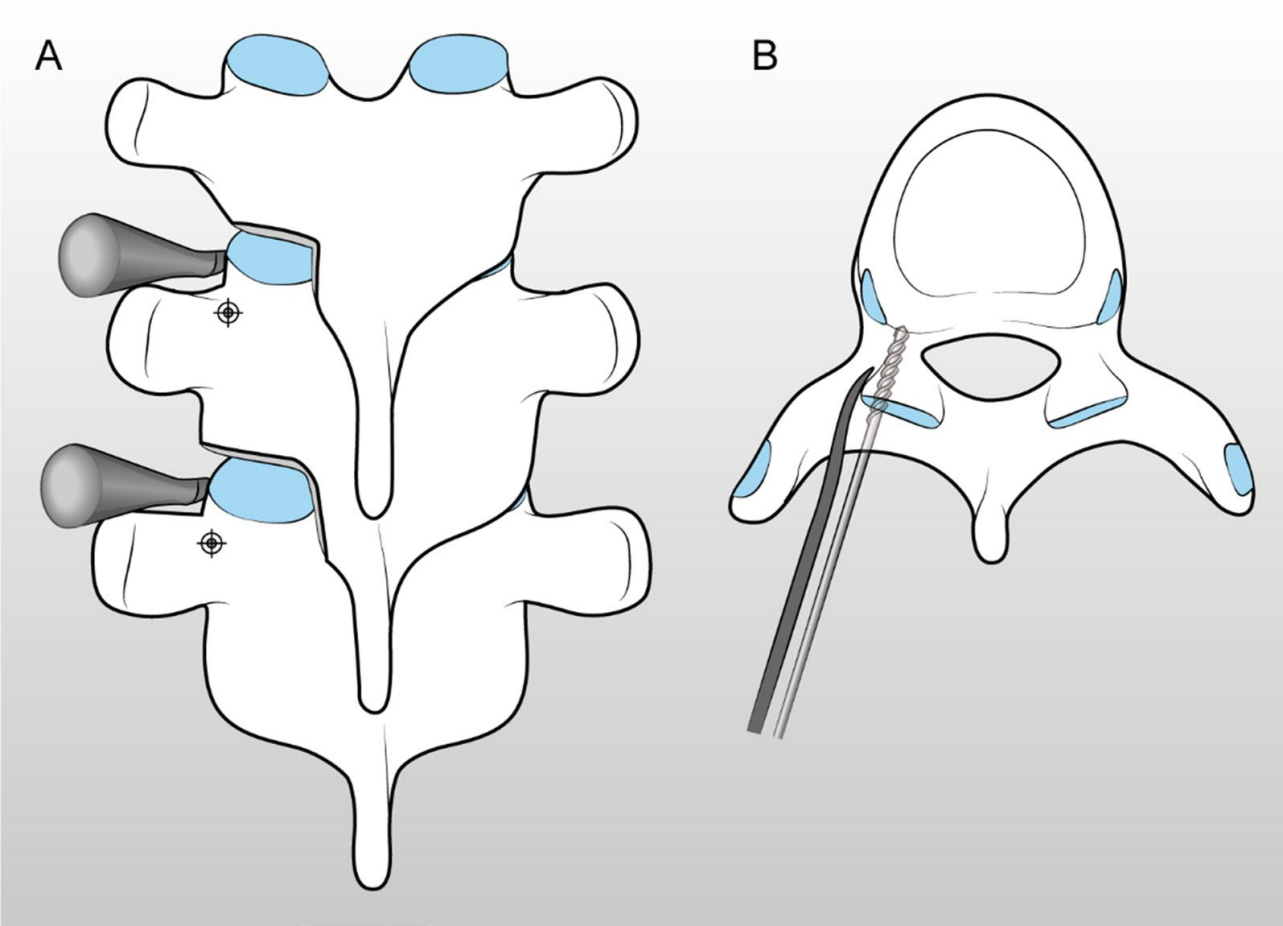
A spatula is placed perpendicular to the superior articular process, serving as a guide for trajectory of the drill bit. Posterior (**A**) and axial (**B**) view

**Fig. 2:**
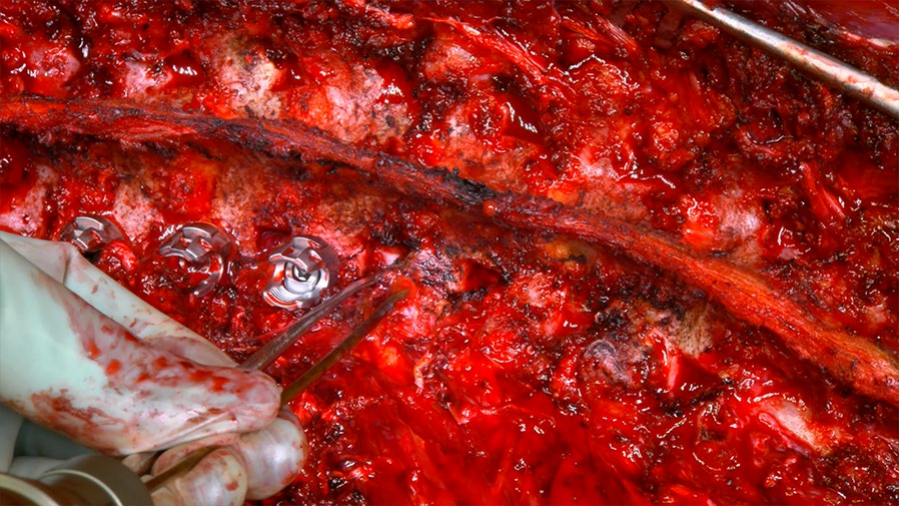
In vivo example of the use of spatula as a guide for drill bit trajectory

After the entry points identification, the cortical bone over it is removed with a rongeur, exposing cancellous bone. This helps the surgeon to find the pedicle and prevents the drill tip from sliding medially on the hard cortex, which may result in an unwanted trajectory deviation. In the thoracic spine, we remove the inferior articular facet with an osteotome, exposing the superior articular process (SAP) which we use as a reference point for pedicle trajectory. In fact, it has been demonstrated that there is a constant angular relationship with the SAP and the pedicle axis: the line perpendicular to the SAP can act as a trajectory [31]. Therefore, we place a spatula perpendicular to the SAP, acting as a guide for trajectory (Figs. 1, 2).

**Fig. 3:**
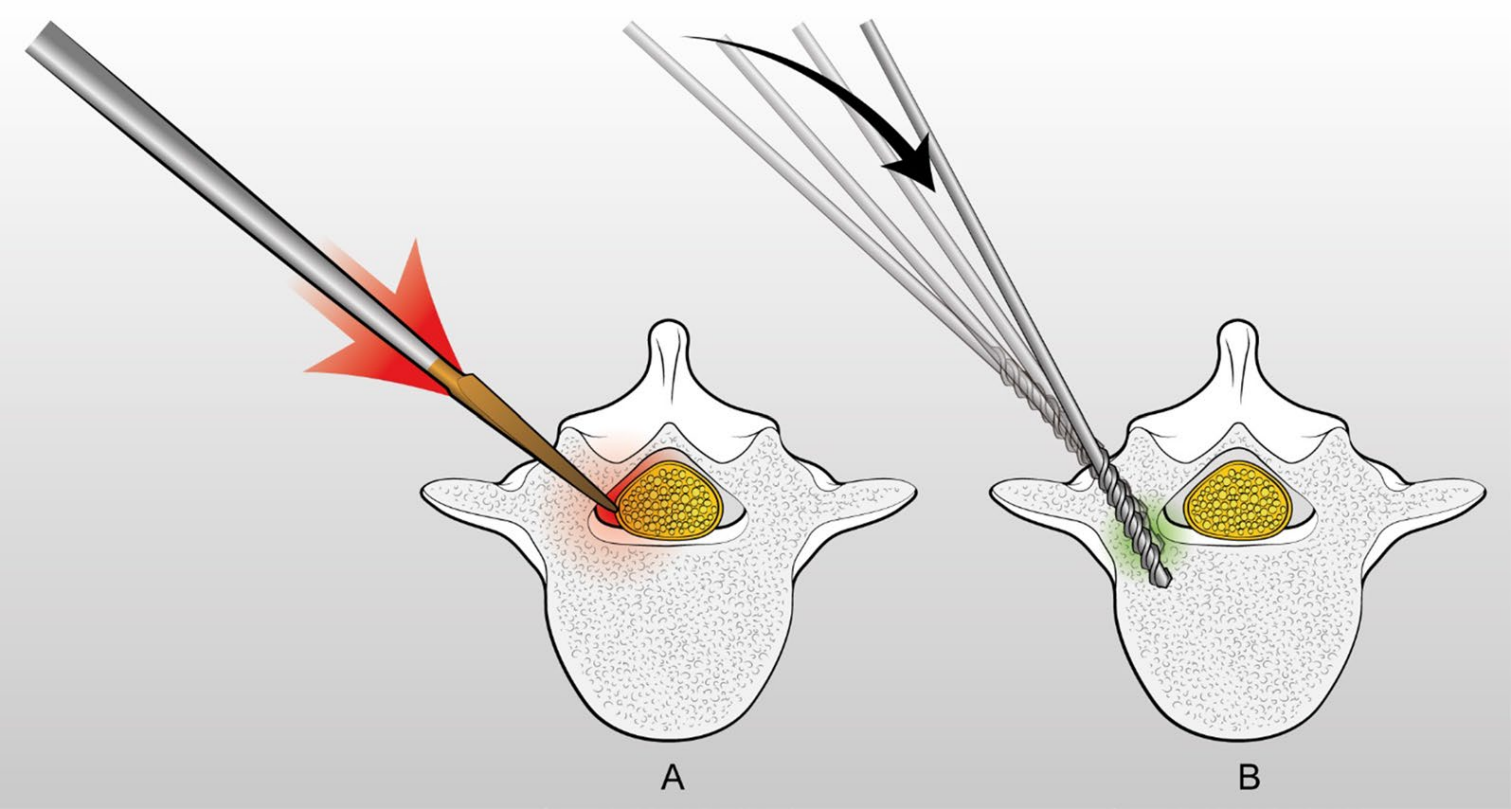
The flexible drill bit (**B**) tends to bend as it encounters hard cortical bone, self-centering into soft cancellous bone, as opposed to the stiffer pedicle probe (**A**)

For pedicle tract preparation, the Authors use a power drill, set in ream mode, with a 2.7 mm tip, following a funnel trajectory. Some others Authors prefer to use a 2 mm bit [18, 23, 32] which, due to its high flexibility, tends to bend as it encounters hard cortical bone, self-centering into soft cancellous bone (Fig. 3). In our opinion, the 2.7 mm diameter is an optimal compromise: it is still flexible enough to bounce off the cortical bone in case of trajectory mistakes, avoiding any breach; at the same time, it is stiff enough to allow to find a way into C and D type of pedicles [33], where, due to the absence of a central cancellous channel, a too much flexible drill bit would bend and would hardly enter. The 2.7 mm drill bit must be slowly rotated (approx. 1–3 rev/s) to optimize the tactile feedback of the threads cutting into the soft cancellous bone. If an increased torque is felt, this means that the drill tip is pointing the pedicle cortex, so small angular corrections may be needed to help the bit to deflect off the cortex into the cancellous pedicle channel. This is the key point of the technique, as the slowly rotating drill bit in experienced hands almost acts like a flexible ball-tipped probe. In fact, one the most frequent mistakes when approaching the power-assisted technique is to rotate the drill too fast, as required by the technique for drilling the long bones, leading to an inevitably loss of any tactile feedback. Additionally, no forward force must be applied, as the weight of the drill is sufficient for the bit to advance into the channel. In fact, as opposed to a smooth pedicle probe, the threads of the drill bit will make it self-advance. As also stated by Illingworth et al. [32], this gives a sensation of the drill bit being “pulled” into the pedicle and provides excellent feedback and confidence that the pedicle has been adequately found. The only situation that requires a fast rotation and a firm pressure of the drill bit is in case of a cortical type C pedicle, where the 2.7 mm drill bit threads have to cut a way into hard cortical bone. In case of a type D pedicle, an in–out-in trajectory may be necessary: the lateral cortex of the pedicle is perforated with a faster rotation of the drill bit and then the cortex of the vertebral body is carefully identified and perforated with the same technique. In case of an in–out-in trajectory, it is important to choose an entry point which is 1–2 mm lateral to the classical one. This allows to maximize the convergence angle, resulting in a double advantage: a stronger pull-out strength [34] and, on the other side, it facilitates the perforation of the lateral vertebral body cortex, avoiding the forward slippage of the drill bit against it. After the pedicle tract preparation, a ball-tip probe is used to rule out any breach and to measure the tract length. If the tract is suspicious for a breach, or the trajectory is unsatisfactory, a new tract can be created without any significant compromise of the pedicle anatomy. In our experience, this is one of main advantages of the drill over the probe. In fact, due to its larger diameter and its less accurate advancing, the probe tends to be less forgiving when creating multiple tracts, resulting in the confluence of the various tracts and ultimately leading in a decreased bone purchase of the screw. Conversely, the drill allows to create distinct, clearly separate, multiple tracts.

After the pedicle tract preparation, we routinely tap the tract, because it reduces the risk of misalignment of the screw with respect to its pilot hole trajectory [35]. We use a 1 mm undertap technique, to optimize the bone-screw purchase. Some Authors [18, 32] prefer to expand the tract so that the pedicle screw can be inserted without tapping; however, it is generally not recommended due to the concern of reduced bone-screw purchase [35]. We generally power tap; however, in case of juxta-pedicular trajectories or poor bone quality, we carry particular attention and use a manual tap, because the sharp grooves could potentially trap neural tissues or lead to wall breaches. We think that creating a tract with a 2.7 mm drill bit, which is larger than 2 mm, may help the tap to follow the existing channel without creating other tracts. After tapping, the tract is reassessed and remeasured with a ball-tipped probe. Finally, a screw of appropriate length and diameter is carefully inserted using a power driver. It is important to keep “soft hands” in order to allow the screw to self-center in the tract with the desired trajectory. The screw should be advanced at low speed, to keep the control of the trajectory and to avoid any breach.

## Conclusions

The literature specifically focusing on power-assisted screw placement is poor and does not provide strength evidence. This literature overview seemed to show that, both in clinical and preclinical studies, the power-assisted technique allows to place screws with an acceptable safety profile, even in the most challenging pedicle anatomies like in AIS. Pedicle screws accuracy seems more surgeon dependent than technique dependent. With adequate training, the power technique may speed up the screw placing and reduce the physical stress to the spine surgeon, making the screw phase more effortless and allowing to save surgeon’s focus for the next, often more critical, surgical steps. Additionally, it could reduce fluoroscopy time, lowering both surgeons’ and patient’s radiation exposure. However, these benefits may not be sufficient to make the switch from the safe and reliable manual technique. The aspect that could really make the difference in clinical outcomes is pull-out strength: unfortunately, information regarding any difference in pull-out strength between power and manual techniques is still uncertain and inconsistent. Therefore, scientific evidence is still not strong enough to guide the surgeon’s choice.
